# Secondary Hepatic Dysfunction in Critically Ill Children: Prognostic Associations Beyond PRISM III and PELOD-2 Scores

**DOI:** 10.3390/jcm15031133

**Published:** 2026-02-01

**Authors:** Tuğba Gürsoy Koca, Abdulkerim Elmas, Ümüt Altuğ, Gürbüz Akçay, Hanife Bayramoğlu, Mustafa Akçam

**Affiliations:** 1 Division of Pediatric Gastroenterology, Hepatology and Nutrition, Department of Pediatrics, Faculty of Medicine, Pamukkale University, Denizli 20160, Turkey; tgkoca@gmail.com; 2 Division of Pediatric Gastroenterology, Hepatology and Nutrition, Department of Pediatrics, Faculty of Medicine, Suleyman Demirel University, Isparta 32260, Turkey; 3 Division of Pediatric Intensive Care Unit, Department of Pediatrics, Faculty of Medicine, Pamukkale University, Denizli 20160, Turkey; drumitaltug@gmail.com; 4 Department of Pediatrics, Faculty of Medicine, Pamukkale University, Denizli 20160, Turkey; 5 Department of Pediatrics, Çerkezköy State Hospital, Tekirdağ 59500, Turkey

**Keywords:** hepatic dysfunction, pediatric intensive care, PRISM score, PELOD-2, sepsis, prognosis

## Abstract

**Background:** Secondary hepatic dysfunction is a frequent yet often under-recognized complication in critically ill children. It commonly arises as a consequence of systemic processes—particularly sepsis, hypoperfusion, hypoxia, and multiorgan dysfunction—rather than primary hepatobiliary disease. This study aimed to determine the incidence, clinical characteristics, and prognostic significance of secondary hepatic dysfunction in a pediatric intensive care unit (PICU) cohort, and to evaluate its relationship with PRISM III and PELOD-2 scores. **Methods:** This retrospective study included patients hospitalized in a tertiary PICU between January 2022 and December 2024. Children with pre-existing liver disease or primary acute liver failure were excluded. Hepatic dysfunction was defined by elevations in age-adjusted biochemical markers. Demographic variables, clinical interventions, laboratory values, and outcomes were recorded. Mortality risk and prolonged PICU stay (>7 days) were analyzed in relation to hepatic dysfunction, PRISM III, and PELOD-2 scores. **Results:** Among 567 PICU admissions, 50 patients (8.8%) met criteria for secondary hepatic dysfunction. The cohort had a median age of 57.5 months and 66% were male. Hepatocellular injury predominated (96%), while cholestatic patterns were less common (4%). Overall mortality was 22%. Mortality was significantly associated with sepsis (*p* = 0.04), mechanical ventilation (*p* < 0.01), and inotropic support (*p* < 0.01). Both PRISM III and PELOD-2 scores were higher in non-survivors on day 1 and day 7 (*p* ≤ 0.01). ALT ≥ 2 × ULN and total bilirubin > 2 mg/dL were not independently predictive of mortality. **Conclusions:** Secondary hepatic dysfunction is relatively common in critically ill children and is associated with adverse clinical outcomes. Its prognostic relevance appears to extend beyond conventional severity scores, particularly with respect to morbidity-related outcomes such as prolonged PICU stay, suggesting that routine hepatic assessment may contribute to early risk stratification in the PICU setting.

## 1. Introduction

Hepatic dysfunction is a common clinical condition in critically ill children, typically arising as a secondary consequence of systemic processes such as sepsis, multiple organ dysfunction, and hemodynamic instability [1,2]. As the liver plays vital roles during critical illness—including maintaining metabolic homeostasis, regulating the immune response, and clearing toxins—its functional impairment can significantly influence the clinical course and prognosis [3]. Therefore, the assessment of liver function is considered an important tool in determining the severity of critical illness.

In patients monitored in the pediatric intensive care unit (PICU), hepatic dysfunction most often develops through secondary mechanisms rather than as a result of primary liver diseases [4]. Hypoperfusion, hypoxia, inflammatory cytokine release, endothelial dysfunction, and drug toxicities are among the primary causes of hepatocellular injury and cholestatic changes in children [5]. Studies in adult intensive care units have reported a strong association between early-onset hepatic dysfunction and both increased mortality and prolonged length of stay in the ICU [1]. However, the higher regenerative capacity of the pediatric liver compared to adults suggests that the role of hepatic dysfunction in prognosis may follow different dynamics in children [6]. It has been emphasized that, in terms of prognosis, the cholestatic pattern is associated with worse outcomes compared to the hepatocellular pattern [2].

Sepsis is one of the most common causes of secondary hepatic dysfunction in pediatric intensive care patients [7]. Systemic inflammation and microcirculatory disturbances that occur during sepsis can lead to reduced hepatic perfusion and impaired bile flow, resulting in liver injury with both hepatocellular and cholestatic patterns [8]. Recent studies have demonstrated that sepsis-associated hepatic dysfunction is significantly associated with increased mortality and prolonged length of stay in the intensive care unit among children [4].

Various clinical scoring systems are used to predict prognosis in critically ill children. The Pediatric Risk of Mortality III (PRISM III) and the Pediatric Logistic Organ Dysfunction Score-2 (PELOD-2) are among the most widely used and validated scores in pediatric intensive care practice [9]. While PRISM III is a mortality prediction tool based on physiological parameters, PELOD-2 assesses the degree of multiple organ dysfunction. Notably, however, liver function parameters are not included in the PELOD-2 scoring system [10]. This omission suggests that the independent prognostic role of hepatic dysfunction in pediatric critical illness remains a subject of debate [11].

The aim of this study is to evaluate the frequency and clinical characteristics of secondary hepatic dysfunction in patients admitted to the pediatric intensive care unit; to examine the relationship between PRISM III and PELOD-2 scores with mortality and length of stay in the ICU; and to investigate the prognostic value of commonly used biochemical parameters such as alanine aminotransferase (ALT) and total bilirubin.

## 2. Methods

This retrospective descriptive study was conducted in patients admitted to the 7-bed Pediatric Intensive Care Unit (PICU) of Pamukkale University Faculty of Medicine Hospital between 1 January 2022, and 31 December 2024. The study was carried out in accordance with the principles of the Declaration of Helsinki and was approved by the institutional ethics committee. (Approval date: 30 September 2025, no: 18).

All patients who were monitored in the PICU for at least 24 h were included in the study. Patients with a known history of chronic liver disease, congenital metabolic disorders, congenital hepatobiliary anomalies, or those admitted with acute liver failure were considered to have primary hepatic dysfunction and were excluded from the study [1,3].

The diagnosis of hepatic dysfunction was based on the presence of at least one liver function test exceeding the age-specific upper limit of normal (ULN). The biochemical tests evaluated included aspartate aminotransferase (AST), alanine aminotransferase (ALT), total bilirubin, direct bilirubin, alkaline phosphatase (ALP), gamma-glutamyl transferase (GGT), international normalized ratio (INR), and serum albumin levels [2]. Age-specific reference ranges were determined based on the *Nelson Textbook of Pediatrics* [12]. Isolated enzyme elevations that could be attributed to extrahepatic causes—such as muscle or bone disorders, disseminated intravascular coagulation (DIC), or malnutrition—were not included in the definition of hepatic dysfunction. Patterns of hepatic dysfunction were classified into two main groups: hepatocellular (predominantly elevated AST and/or ALT) and cholestatic (predominantly elevated total/direct bilirubin, ALP, and/or GGT). Secondary hepatic dysfunction was defined using conventional biochemical markers, including alanine aminotransferase (ALT) and total bilirubin levels. An ALT level ≥ 2 times the upper limit of normal (ULN) and/or total bilirubin > 2 mg/dL were selected to reflect clinically relevant hepatic involvement rather than transient biochemical abnormalities, consistent with definitions used in pediatric critical care and organ dysfunction literature [11,13].

The definitions of sepsis and sepsis-related organ dysfunction were based on the criteria of the International Pediatric Sepsis Consensus [7]. Hypoxia was defined as a partial pressure of arterial oxygen to fractional inspired oxygen ratio (PaO_2_/FiO_2_) of less than 300 mmHg [14].

Demographic characteristics, admission diagnoses, occurrence of sepsis during the clinical course, need for mechanical ventilation, use of inotropic support, total parenteral nutrition (TPN), blood product transfusions, and administered treatments (including antibiotics, antiepileptics, and N-acetylcysteine) were retrospectively obtained from patient files and electronic medical records.

Disease severity and the extent of organ dysfunction were assessed using the PRISM III and PELOD-2 scoring systems. While the PRISM III score estimates mortality risk based on physiological parameters, the PELOD-2 score evaluates the degree of multiple organ dysfunction [9,10]. Due to the absence of liver function parameters in the PELOD-2 scoring system, the prognostic impact of hepatic dysfunction was analyzed separately, independent of these scoring systems. Both PRISM III and PELOD-2 scores were calculated within the first 24 h of PICU admission and again on the 7th day of hospitalization.

Statistical analyses were performed using SPSS software (version 27.0; IBM Corp., Armonk, NY, USA). Continuous variables were presented as mean ± standard deviation (SD) or median [interquartile range; IQR], depending on distribution characteristics, while categorical variables were reported as numbers and percentages (%). The Mann–Whitney U test was used for comparisons of continuous variables due to non-normal distribution. The Chi-square (χ^2^) test or Fisher’s exact test, when appropriate, was applied for comparisons of categorical variables. For potential risk factors associated with secondary hepatic dysfunction and mortality, odds ratios (ORs) and 95% confidence intervals (CIs) were calculated. A multivariable logistic regression analysis was performed to evaluate factors independently associated with mortality. Mortality was entered as the dependent variable. ALT, total bilirubin, PRISM III score on day 1, and PELOD-2 score on day 1 were included as independent variables. Results were expressed as odds ratios (ORs) with 95% confidence intervals (CIs). A *p*-value of <0.05 was considered statistically significant.

## 3. Results

During the study period, secondary hepatic dysfunction was identified in 50 out of 567 patients (8.8%) monitored in the PICU. The median age of the cases was 57.5 months (IQR: 18.5–156.2), and 33 patients (66.0%) were male. The most common reason for admission was respiratory diseases (*n* = 23, 46.0%). Other admission diagnoses included sepsis (*n* = 8, 16.0%), neurological disorders (*n* = 6, 12.0%), multiple trauma (*n* = 6, 12.0%), nephrological conditions (*n* = 6, 12.0%), and primary liver disease (*n* = 1, 2.0%).

The predominant pattern of hepatic dysfunction was hepatocellular (*n* = 48, 96.0%), while a cholestatic pattern was observed in only two cases (4.0%). The overall mortality rate was 22.0% (*n* = 11).

Sepsis was observed in 22 patients (44.0%). Four patients (8.0%) received total parenteral nutrition (TPN), with a mean duration of 10.5 days. Findings related to patients’ age, sex, PRISM III and PELOD-2 scores, presence of sepsis, intensive care support measures, administered treatments, length of stay, mortality, and liver enzyme levels are presented in Table 1.

PRISM III and PELOD-2 scores were assessed on both the first and seventh days of admission. In both scoring systems, scores were significantly higher in patients who died compared to survivors (Table 2). On day 1, PRISM III and PELOD-2 were higher in the mortality group (*p* = 0.001 and *p* < 0.01, respectively), and this difference persisted on day 7 (*p* = 0.013 and *p* = 0.001, respectively; Table 2).

Analysis of clinical factors associated with mortality revealed that sepsis, mechanical ventilation, and inotropic support were significantly associated with mortality (*p* = 0.04, *p* < 0.01, and *p* < 0.01, respectively) (Table 3). No statistically significant associations were found between mortality and the use of TPN, blood product transfusions, antibiotics, antiepileptic treatment, or N-acetylcysteine (NAC).

In a multivariable logistic regression model including ALT, total bilirubin, PRISM III (day 1), and PELOD-2 (day 1), neither ALT nor total bilirubin remained independently associated with mortality after adjustment for severity scores. Among the severity indices, PELOD-2 showed a borderline association with mortality (OR: 1.126, 95% CI: 0.999–1.269, *p* = 0.051), whereas PRISM III was not significant.

A prolonged PICU stay (>7 days) was significantly associated with sepsis, mechanical ventilation, inotropic support, blood product transfusion, and antibiotic therapy (Table 4). In univariate comparisons, PRISM III (day 1) was higher in patients with prolonged stay (*p* = 0.03), whereas PELOD-2 (day 1) did not differ significantly between groups (*p* = 0.14) (Table 4).

In the subgroup analysis based on ALT and total bilirubin threshold values, no statistically significant association was found between mortality and either ALT ≥ 2 × ULN or total bilirubin > 2 mg/dL. Patients with ALT ≥ 2 × ULN had a shorter PICU length of stay compared with those with ALT < 2 × ULN (Table 5).

In multivariable logistic regression analysis for prolonged PICU stay (>7 days), ALT level showed a borderline independent association after adjustment for PRISM III and PELOD-2 scores (OR: 0.996, 95% CI: 0.991–1.000, *p* = 0.051). Total bilirubin was not independently associated with prolonged hospitalization (*p* = 0.951). Among severity scores, PRISM III demonstrated a borderline association (OR: 1.149, 95% CI: 0.990–1.334, *p* = 0.068), whereas PELOD-2 was not significant (*p* = 0.122).

## 4. Discussion

This study demonstrated that secondary hepatic dysfunction is commonly observed in children admitted to the PICU and is associated with adverse clinical outcomes. When disease severity was taken into account, conventional hepatic biochemical markers did not independently predict mortality. Instead, hepatic involvement appeared to be more closely related to morbidity-related outcomes, particularly prolonged PICU stay. These findings support the notion that hepatic dysfunction in critically ill children more often reflects the systemic effects of critical illness rather than primary liver disease, highlighting the importance of early assessment of overall organ dysfunction burden in the PICU setting.

The frequency of hepatic dysfunction observed in our study is consistent with previous pediatric intensive care reports. Prior studies have reported secondary hepatic dysfunction rates ranging from approximately 12% to 30% in PICU patients, particularly among children with sepsis or multiple organ dysfunction, and have linked hepatic injury—especially cholestatic patterns—to prolonged PICU stay, increased organ support requirements, and higher mortality [4,13,15,16,17,18]. In line with this literature, we observed an incidence of secondary hepatic dysfunction of 8.8% in our cohort, supporting the clinical relevance of hepatic involvement in critically ill children, particularly as a marker of disease burden and morbidity [19,20].

In sepsis, the liver acts as both a target and a central regulator of the systemic inflammatory response, contributing to inflammatory mediator clearance, acute-phase and complement activation, coagulation factor synthesis, and detoxification [3,21]. Sepsis-associated hepatic dysfunction has been identified as an independent risk factor for multiple organ dysfunction and mortality [2,3,8,21,22]. Cholestatic patterns in critical illness arise from the combined effects of inflammatory cytokines, endotoxins, hypoperfusion, hypoxia, and drug toxicity, reflecting the severity of systemic inflammation [22,23]. In line with this pathophysiology, our study demonstrates a strong association between secondary hepatic dysfunction, sepsis, and multiple organ dysfunction, suggesting that liver impairment reflects—and may amplify—the systemic inflammatory burden [3,21].

In this context, it is important to distinguish acute liver failure from secondary hepatic dysfunction. According to the PODIUM consensus, acute liver dysfunction represents an intermediate phenotype in critically ill children, characterized by abnormalities in bilirubin and coagulation parameters without fulfilling classic acute liver failure criteria [11]. This phenotype most commonly develops in the setting of sepsis and multiple organ dysfunction and differs clinically and prognostically from primary acute liver failure caused by viral infections or drug toxicity [11,19,20]. Even overt pediatric acute liver failure follows a heterogeneous course in intensive care, limiting reliable prognostic stratification [20,24]. These observations underscore the need to assess liver involvement in pediatric critical illness across a broader continuum, within which secondary hepatic dysfunction represents a key component [11,20,24].

In our study, conventional hepatic biochemical markers did not independently predict mortality after adjustment for PRISM III and PELOD-2 scores. Although these severity scoring systems are widely used to provide global assessments of physiological derangement and organ dysfunction, neither PRISM III nor PELOD-2 independently predicted prolonged PICU stay in multivariable analysis. This finding suggests that prolonged hospitalization reflects dimensions of disease burden not fully captured by existing severity scores. In this context, hepatic dysfunction may provide complementary information regarding morbidity and resource utilization rather than acting as an independent determinant of survival. Hepatic dysfunction has been described as an “overlooked organ dysfunction” in pediatric intensive care, as it is insufficiently captured by current scoring systems [24]. Consistent with this perspective, our findings suggest that incorporating secondary hepatic dysfunction alongside established severity scores may enhance risk stratification and facilitate earlier identification of high-risk pediatric patients [16,18,24,25].

In our study, ALT ≥ 2 × ULN and total bilirubin > 2 mg/dL were not significantly associated with mortality, suggesting that conventional biochemical thresholds alone may have limited prognostic value. Adult intensive care studies indicate that prognosis is influenced not only by the degree of hepatic biochemical derangement but also by its duration, pattern (cholestatic vs. hepatocellular), and coexistence with other organ dysfunctions [1,2,6,22,23]. Pediatric studies have yielded heterogeneous results, with some reporting associations between elevated liver enzymes and prolonged PICU stay or mortality, while others have found weaker or non-significant relationships [4,13,15]. Early enzyme elevation has been proposed as a risk marker when combined with clinical parameters; however, in our cohort, low mortality among patients with mild to moderate ALT and bilirubin elevations suggests limited discriminative power of these thresholds in isolation [17]. In contrast, in our cohort, the observation of low mortality among some patients with only mild to moderate elevations in ALT and bilirubin suggests that these thresholds may not possess sufficient discriminative power on their own in heterogeneous populations. Such discrepancies likely reflect differences in study design, etiological profiles, intensive care practices, and selected cut-off values [13,15,16,17,18].

In recent years, studies focusing on biomarkers and ratios that go beyond conventional liver function tests have gained attention in the evaluation of secondary hepatic dysfunction. Notably, total bile acid levels have been reported to be associated with multiple organ dysfunction and mortality in pediatric sepsis, potentially offering superior prognostic performance compared to traditional aminotransferase and bilirubin measurements [5]. The bilirubin-to-albumin ratio has also emerged as a promising parameter for predicting intensive care mortality in both adult and pediatric populations [16,26,27]. Kang et al. demonstrated that the bilirubin-to-albumin ratio was associated with mortality in children admitted to the PICU, particularly among those requiring respiratory support and vasopressors. They suggested that this ratio could serve as a practical and easily applicable tool for risk stratification [28]. In adult intensive care and liver cirrhosis cohorts, the bilirubin-to-albumin ratio has consistently been shown to have a strong association with short- and medium-term mortality [26,27]. Although such ratios or bile acid–based biomarkers could not be evaluated in our study, we believe that incorporating these novel parameters alongside conventional liver tests in future prospective studies may provide a more nuanced understanding of secondary hepatic dysfunction phenotypes and improve risk stratification [5,16,26,27].

The clinical management implications of secondary hepatic dysfunction are also significant. In cases where cholestatic patterns or coagulopathy-associated hepatic dysfunction arise, particularly in the context of sepsis-induced multiple organ dysfunction, careful attention to fluid management, titration of vasopressors/inotropes, selection of sedatives and antibiotics, nutritional strategies, and avoidance of potentially hepatotoxic medications plays a critical role in preventing further deterioration of liver function [1,3,6,22,23]. In our study, the higher rates of mechanical ventilation, inotropic support, blood product transfusion, and antibiotic therapy among children with hepatic dysfunction highlight the intricate interplay between hemodynamic and respiratory instability and liver involvement. This underscores the importance of regular and dynamic monitoring of liver function tests in the PICU, prompt reinterpretation of evolving values within the clinical context, and, when necessary, the adoption of a multidisciplinary approach—including hepatology consultation—to optimize patient management [11,16,24,25].

Although hepatocellular injury predominated in our cohort, with cholestatic patterns observed less frequently, the recent literature has highlighted severe forms of cholestatic liver dysfunction developing in selected critically ill pediatric populations. In particular, secondary sclerosing cholangitis in critically ill patients has been increasingly recognized as a distinct and severe phenotype, especially in the context of febrile infection-related epilepsy syndrome (FIRES). A recent report described progressive cholestatic liver injury and biliary tract damage in critically ill children with FIRES, emphasizing the role of prolonged systemic inflammation, ischemia, and intensive care–related factors in the pathogenesis of this condition [29]. These observations underscore the heterogeneity of secondary hepatic dysfunction in pediatric critical illness and suggest that cholestatic phenotypes may be underrepresented in general PICU cohorts but clinically relevant in specific high-risk settings. Heightened clinical awareness and tailored diagnostic strategies may therefore be warranted in selected patient populations.

The main limitations of this study include its single-center, retrospective design and relatively small sample size, which may limit generalizability and statistical power. Comprehensive liver evaluations could not be uniformly performed in all patients in the acute critical care setting, and mild underlying chronic liver or metabolic diseases may not have been fully excluded, potentially leading to underestimation of secondary hepatic dysfunction. In particular, subclinical or previously undiagnosed liver diseases with an insidious course, such as autoimmune hepatitis, may have become clinically apparent during sepsis, intercurrent infection, or drug exposure; however, standardized diagnostic work-ups, including systematic autoantibody screening and application of validated autoimmune hepatitis diagnostic scoring systems, could not be consistently applied due to the retrospective design [30]. In addition, conventional hepatic biochemical markers were used, and comparisons with emerging or composite biomarkers—such as the bilirubin-to-albumin ratio or bile acid–based parameters—were not performed; these novel biomarkers may better reflect dynamic hepatic dysfunction and could provide additional prognostic value. Furthermore, longitudinal trends of hepatic biomarkers and their temporal relationship with clinical outcomes could not be evaluated. Nevertheless, the extended study period, systematic review of intensive care records, and use of validated severity scores such as PRISM III and PELOD-2 strengthen the reliability of our findings.

In conclusion, secondary hepatic dysfunction is common among children admitted to the PICU and is strongly associated with mortality, independent of global severity scores. Conventional biochemical thresholds alone appear insufficient for mortality prediction, as disease severity is more closely driven by sepsis and multiple organ dysfunction. Systematic monitoring of hepatic dysfunction may enhance risk stratification and inform future biomarker development. Large-scale, multicenter prospective studies integrating novel biomarkers alongside established severity scores are warranted to better define the prognostic role of secondary hepatic dysfunction in critically ill children.

## Figures and Tables

**Table 1:** Demographic and clinical characteristics of patients with secondary hepatic dysfunction (*n* = 50).

Variable	Value
Age (months), median (IQR)	57.5 (18.5–156.2)
Sex, male, *n* (%)	33 (66.0)
PRISM III score (Day 1), median (IQR)	6 (2.7–19.2)
PELOD-2 score (Day 1), median (IQR)	10 (0–21)
Sepsis, *n* (%)	22 (44.0)
Mechanical ventilation, *n* (%)	32 (64.0)
Inotropic therapy, *n* (%)	24 (48.0)
Total parenteral nutrition (TPN), *n* (%)	4 (8.0)
Blood product transfusion, *n* (%)	27 (54.0)
Antibiotic therapy, *n* (%)	46 (92.0)
Antiepileptic therapy, *n* (%)	26 (52.0)
N-acetylcysteine (NAC) therapy, *n* (%)	2 (4.0)
Length of stay in the intensive care unit (days), median (IQR)	10 (4.7–31.2)
Mortality, *n* (%)	11 (22.0)
AST, median (IQR)	104 (49.5–187)
ALT, median (IQR)	91.5 (31.7–153.7)

Data are presented median IQR (interquartile range) (25th and 75th percentiles). Abbreviations: PRISM III, Pediatric Risk of Mortality Score; PELOD-2, Pediatric Logistic Organ Dysfunction Score; NAC, N-acetylcysteine; AST, aspartate aminotransferase; ALT, alanine aminotransferase.

**Table 2:** Comparison of PRISM III and PELOD-2 scores between survivors and non-survivors.

Score	Survivors (*n* = 39)	Exitus (*n* = 11)	*p*
PRISM III (Day 1), median (IQR)	5 (2–11)	21 (14–27)	**0.001**
PELOD-2 (Day 1), median (IQR)	2 (0–11)	31 (12–42)	**<0.01**
PRISM III (Day 7), median (IQR)	6 (0–15)	21.5 (11–28)	**0.012**
PELOD-2 (Day 7), median (IQR)	10 (0–12)	31 (18.7–41.2)	**<0.01**

Data are presented median IQR (interquartile range) (25th and 75th percentiles). The Mann–Whitney U test was used for comparisons between groups. Abbreviations: PRISM III, Pediatric Risk of Mortality Score; PELOD-2, Pediatric Logistic Organ Dysfunction Score. Bold values indicate statistically significant results (*p* < 0.05).

**Table 3:** Association between clinical factors and mortality.

Clinical Factor	Survivors (*n* = 39)	Exitus (*n* = 11)	*p*
Sepsis, *n* (%)	14 (35.9)	8 (72.7)	0.04 *
Mechanical ventilation, *n* (%)	21 (53.8)	11 (100)	<0.01 *
Inotropic therapy, *n* (%)	13 (33.3)	11 (100)	<0.01 *
Total parenteral nutrition (TPN), *n* (%)	2 (5.1)	2 (18.2)	0.2 *
Blood product transfusion, *n* (%)	22 (56.4)	5 (45.5)	0.73 *
Antibiotic therapy, *n* (%)	36 (92.3)	10 (90.9)	NA
Antiepileptic therapy, *n* (%)	20 (51.3)	6 (54.5)	NA
N-acetylcysteine (NAC) therapy, *n* (%)	2 (5.1)	0 (0)	NA

Data are presented as number (percentage). Comparisons between groups were performed using the Chi-square test or Fisher’s exact test (*) when cell frequencies were low. Abbreviations: NA: not applicable. Bold values indicate statistically significant results (*p* < 0.05).

**Table 4:** Factors associated with prolonged PICU length of stay (>7 days).

Variable	Short LOS (≤7 Days) *n* = 20	Long LOS (>7 Days) *n* = 30	*p*
PRISM III (Day 1), median (IQR)	2.5 (0–17)	8 (4–20.2)	**0.03**
PELOD-2 (Day 1), median (IQR)	0.5 (0–26)	11 (1.7–21)	0.14
PRISM III (Day 7), median (IQR)	2 (0–2)	9.5 (1–19.2)	0.17
PELOD-2 (Day 7), median (IQR)	10 (0–10)	11 (0–21)	0.22
Sepsis, *n* (%)	3 (15)	19 (63.3)	<0.01 *
Mechanical ventilation, *n* (%)	8 (40)	24 (80)	**<0.01**
Inotropic therapy, *n* (%)	6 (30)	18 (60)	**0.03**
Blood product transfusion, *n* (%)	6 (30)	21 (70)	**<0.01**
Antibiotic therapy, *n* (%)	16 (80)	30 (100)	0.02 *
Total parenteral nutrition (TPN, *n* (%)	0 (0)	4 (13.3)	0.14 *
AST (IU/L), median (IQR)	123.5 (38–396)	97 (49–162)	0.51
ALT (IU/L), median (IQR)	112 (56–299)	60 (23–128)	**0.02**
Total bilirubin (mg/dL), median (IQR)	0.37 (0.24–0.69)	0.42 (0.24–0.8)	0.95
Antiepileptic therapy, *n* (%)	8 (40)	18 (60)	0.16
N-acetylcysteine (NAC) therapy, *n* (%)	1 (5)	1 (3.3)	NA

Data are presented median IQR (interquartile range) (25th and 75th percentiles) or number (percentage). Comparisons between groups were performed using the Mann–Whitney U test for continuous variables and the Chi-square test or Fisher’s exact test (*) for categorical variables. Abbreviations: PRISM III: Pediatric Risk of Mortality Score; PELOD-2: Pediatric Logistic Organ Dysfunction Score, LOS: Length of Stay, NA: not applicable. Bold values indicate statistically significant results (*p* < 0.05).

**Table 5:** Association of ALT and total bilirubin levels with mortality and length of stay.

Parameter	Group	Mortality (*n*, %)	OR (95% CI)	*p*	Length of Stay, (Days) Median (IQR)	*p*
ALT	<2 × ULN (*n* = 24)	4 (16.7)	-		15 (7.2–35.7)	
	≥2 × ULN (*n* = 26)	7 (26.9)	1.75 (0.47–6.58)	0.501	7 (3–15.2)	**0.04**
Total bilirubin	≤2 mg/dL (*n* = 48)	9 (22.5)	-		12 (6–35)	
	>2 mg/dL (*n* = 2)	0	0.65 (0.03–14.59)	1.00	3.5 (1–6)	0.09

Data are presented median IQR (interquartile range) (25th and 75th percentiles) or number (percentage). Comparisons between groups were performed using Fisher’s exact test. Abbreviations: ALT, alanine aminotransferase; ULN, upper limit of normal; OR, odds ratio; CI, confidence interval. Bold values indicate statistically significant results (*p* < 0.05).

## Data Availability

The original contributions presented in this study are included in the article. Further inquiries can be directed to the corresponding author.
